# Mortality Resulting from Undesirable Behaviours in Dogs Aged Three Years and under Attending Primary-Care Veterinary Practices in Australia

**DOI:** 10.3390/ani11020493

**Published:** 2021-02-13

**Authors:** Yan Yu, Bethany Wilson, Sophie Masters, Diane van Rooy, Paul D. McGreevy

**Affiliations:** 1 Sydney School of Veterinary Science, Faculty of Science, University of Sydney, Sydney, NSW 2006, Australia; bethany.wilson@sydney.edu.au (B.W.); sophie.masters@sydney.edu.au (S.M.); paul.mcgreevy@sydney.edu.au (P.D.M.); 2 School of Life and Environmental Sciences, Faculty of Science, University of Sydney, Sydney, NSW 2006, Australia; diane.vanrooy@sydney.edu.au

**Keywords:** animal welfare science, canine behaviour, VetCompass Australia

## Abstract

**Simple Summary:**

There is increasing evidence that undesirable behaviours (UBs) in dogs can compromise the welfare of both canine companions and their associated humans. Indeed, in a recent UK study of patient records from primary-care veterinary practices, UBs emerged as the predominant cause for mortality in young dogs. The current companion study of dogs attending veterinary practices in Australia from 2013 to 2018 reports a comparable proportion of mortality (29.7%) due to UBs among dogs aged three years and under. The most commonly reported UB was aggression. Neutered dogs and purebred dogs (and specifically Australian Cattle Dogs and American Staffordshire terriers) had an elevated risk of death ascribed to at least one UB. The risk factors associated with these UBs are also reported, including interventions applied by the attending clinician (if any). The results highlight the influence of UBs on dog welfare in Australia, and infer the benefits of educating dog owners and veterinary professionals in modifying and managing UBs.

**Abstract:**

There is increasing evidence that undesirable behaviours (UBs) in dogs can compromise the welfare of both canine companions and their carers. Veterinarians are regularly consulted about affected animals and may be asked to euthanase the more severely affected individuals. A recent study of veterinary records showed that UBs were the predominant cause of mortality in young dogs in the UK. This companion study from Australia reports the proportion of mortality due to UBs among dogs aged three years and under that attended veterinary practices from 2013 to 2018. Deidentified patient records were extracted from the VetCompass Australia database and manually assessed to reveal the prevalence and type of UBs reported. The results reveal that 29.7% of the 4341 dogs that died at three years of age or under had deaths ascribed to at least one UB, and that the most commonly reported UB was aggression. Neutered dogs had 2.5× the odds of death due to an UB compared to intact dogs, and crossbred dogs were found to have 1.43× the odds of a UB related death compared to purebred dogs. The breeds at highest risk were Australian cattle dogs (odds ratio (OR) 4.77) and American Staffordshire terriers (OR 4.69). The attending veterinarian referred behaviour cases to a behaviourist or dog trainer in 11.0% of all UB cases, and attempted pharmacological therapy in 5.9% of cases. The results reveal how often UBs affect dogs and their owners in Australia, and infer the beneficial impact that educating dog owners and veterinary professionals in modifying and managing UBs may have.

## 1. Introduction

Australia has one of the highest rates of companion dog ownership in the world, with 40% of households reporting the presence of at least one dog [1]. Whilst most owners consistently state that their dogs enrich their lives, some have preconceived expectations concerning canine behaviour that their dogs fail to meet [2,3]. Unmet and unrealistic expectations can contribute to conflict between dogs and their owners, which increases the risk of those dogs undergoing neglect, abandonment, and euthanasia.

Undesirable behaviours (UBs) in dogs include any unwanted response as reported by associated humans, be they the owner, a neighbour, or veterinary staff. They can present in the form of a behavioural pathology, such as stereotypic tail-chasing, manifest as a consequence of physiological or medical dysfunction, such as inappropriate elimination occurring as a symptom of a urinary tract infection, or simply be an expression of normal canine behaviour such as vocalisation [4]. Risk factors for UB have been previously reported, and include breed [5,6,7,8], selective breeding for temperament and genetics [9,10,11], age [12,13], coupled with a wide variety of environmental factors including mother–young relationship [14], prenatal environment [15], early experience [16,17], and adequate exposure to mental stimulation and socialisation [4,15]. In addition, recent studies have also shown that age at neutering and neuter status [13] influence numerous associated behaviours in both male [10] and female [18] dogs.

Whether a behaviour is viewed as desirable or undesirable is determined solely by the humans in the human–dog dyad. Elevated reporting of UB in dogs can occur when there is mismatch between owner and dog personality [19], or when the dog’s behaviour does not fulfil the owner’s original reasons for wanting a dog [20]. The presence of multiple UBs correlates negatively with the amount of time owners spend with their canine companion [21], and suggests that owners can develop negative feelings towards their dogs [22]. Dissatisfaction and negative emotions arising from UBs can weaken the human–dog attachment bond, and increase the risk of punishment or neglect of the dog [23]. Owners’ approaches to UBs can even infer issues with owners’ mental health. For example, it has recently been shown that men with moderate depression were five times more likely to use punitive training methods than women without depression, and that their dogs were more likely to show familiar dog aggression and house-soiling compared to the median score for dogs in that study [24]. The authors of that study postulated that the UBs may have been either the cause or result of aversive training [24]. Other sources of friction may arise when an owner’s reason for acquiring a dog are at odds with the behavioural needs of the selected breed [24,25], leading to mismatches in housing, socialisation, and physical activity requirements [26].

The type of UB may also affect outcomes for the dog. Among dogs with UBs, those exhibiting aggressive behaviours are at the highest risk of euthanasia [27]. Anxiety is one of the other most common behaviours that compromises the relationship owners have with their dogs, commonly presenting in the form of UB(s) such as excessive vocalisation, destructive behaviour, and inappropriate elimination [28]. A weakened human–dog bond also increases the risk of abandonment, surrender, and mortality of domestic dogs [29]. The latest annual report (2018–2019) by the Royal Society for the Prevention of Cruelty to Animals (RSPCA) Australia showed that, of the total dogs euthanased through their shelters, 67.5% were due to behavioural reasons [30]. Reports of UBs resulting in abandonment and death of companion dogs have been repeated worldwide [31,32,33]. Such outcomes have a significant harmful impact on the welfare of dogs and their carers, including but not limited to dog owners, veterinary, and shelter staff [34,35]. The management of unwanted dogs is also a significant source of municipal and welfare agency spending [36].

Boyd et al. [37] reported that among 1574 dogs attending primary care veterinarians in the United Kingdom (UK), the expression of UBs was the predominant reason for death in dogs under three years of age (33.7%). Their report was based on information extracted from clinics through the UK’s VetCompass database. The current study aims to add an Australian perspective by producing a comparable analysis of UBs as a cause for mortality in dogs aged three years and under, using data from VetCompass Australia.

## 2. Materials and Methods

### 2.1. VetCompass Australia Recruitment Criteria

VetCompass Australia (VCA) is a nationwide surveillance system that collects veterinary medical records from first opinion (primary care) practices across the country (*n* = 180) and shares the collated data with all seven Australian veterinary schools [38]. The results of a previous demographic study [39] suggest that the caseload of VetCompass clinics is representative of the wider Australian population of cats and dogs that attend veterinary clinics. The current study used deidentified data from electronic patient records (EPR) collected over the period of 1 January 2013 to 21 August 2018 to report on an estimated proportional mortality and associated risk factors for mortality from UB in dogs that died aged three years and under, using a cross-sectional study design. The initial sampling criterion specified that dogs had to have had their record deactivated at the age of three years or younger. Other patient demographic data included: patient breed; colour; sex; insurance status; neuter status; and bodyweight. Clinical information included free-text for each consultation, the date of the consultation, and the state or territory from which the information was derived. The depth and detail of information recorded in the consultation text were highly variable, reflecting the range of reporting styles of clinicians, and the software and protocols employed by clinics.

### 2.2. Data Curation

Data were manually evaluated for consistency, missing values, duplicates, and outliers through Excel^®^ (Microsoft Office Excel^®^ 2017, Redmond, WA, USA). Dogs were recorded as deactivated for the following reasons: no reason was explicitly given; confirmed death of the animal; patient lost to follow up; or the owner had relocated and no longer attended the participating clinic. The EPR of each dog was reviewed directly to identify those that had truly died. Of those, the cause, date, mechanism of death, and whether the cause of death was related to the dog expressing any UBs was ascertained. Records were omitted from the final analysis if the EPR met any of the following exclusion criteria: the examination text field was empty; or death was not explicitly stated as the reason for the discontinuation of the record. The remaining records were assigned to the following three categories: UB-related death, where cause of death was ascribed to the dog exhibiting one or more UBs; non-UB related death; and not specified, where the examination text confirmed death without any additional information. Non-UB-related deaths included: illness, which includes toxic, congenital, and infectious causes; and miscellaneous reasons for euthanasia, such as the owner no longer being able to care for the dog. Mechanism of death was also recorded in three categories: assisted, where death occurred through euthanasia at a veterinary clinic; unassisted, such as when the patient was dead on arrival upon presenting to the clinic, died whilst treatment was attempted, or confirmed through client communication after the fact; and not specified, where patient death was recorded in the clinical notes but no additional information was provided.

Definitions, inclusion and exclusion criteria, disorder categories, and classification of variables were consistent with Boyd et al. [37]. “UB associated mortality” was defined as the EPR explicitly attributing the cause of death to the dog expressing at least one of the following: one or more UB(s); involved in a road traffic accident (RTA) as the primary cause of death; or due to another dog exhibiting an UB (for example, if the case dog died due to a dog bite wound). A UB was determined as any behavioural attribute recorded in the EPR that was stated as being unwelcomed to the owner and/or others. Dogs displaying UBs were allocated into 16 UB disorder categories (File S1). All individual UB disorder terms mentioned in the EPR were counted and ranked according to frequency of deaths involving that UB disorder term. For example, if a dog exhibited three different UBs, the count of each respective UB category would increase by one.

The following information where available was also recorded for any dogs whose death was ascribed to UB(s): any medical or behavioural intervention prescribed by the veterinarian or sought out by the owner prior to euthanasia, including any attempts at rehoming; the age at which the case dog first displayed an instance of UB prior to death; the age of gonadectomy; contributory reason regarding why gonadectomy was performed; and the acquisition source of the dog. Among acquisition source terms, “backyard breeder” was attributed to individual dogs only where the term was explicitly mentioned in the EPR to describe the dog’s source of acquisition.

### 2.3. Variables

A purebred and crossbred variable was established according to VetCompass Australia breed groupings (File S2), which grouped any dogs with a singular recognisable breed entered in the EPR breed field as purebred, and classified all other dogs as crossbred. This included any dog that had a primary breed identifier in their breed field, for example “Great Dane X”. Any breed that contained fewer than 15 individual dogs were grouped together into an “Other Purebred” category for regression analysis, corresponding with the method in Boyd et al. [37]. Breed groups were established based on groupings recognised by the Kennel Club (KC): gundog; hound; non-sporting; terrier; toy; utility; and working. All remaining dogs were classified as non-KC recognised. “Age” described the age at death (years) and was categorised into three groups (<1.0, 1.0 < 2.0, and 2.0– ≤ 3.0). “Bodyweight” (kg) described the maximum bodyweight recorded during the study period and was categorised into six groups (0.0–9.9, 10.0–19.9, 20.0–29.9, 30.0–39.9, ≥40.0, and not recorded). Sex, neuter status, and insurance status were allocated to each dog according to what was entered in the last EPR.

### 2.4. Data Analysis

Where possible the following analysis aimed to replicate the methodology as outlined in Boyd et al. [37]. Of the dogs that died with an ascribed reason, proportional mortality and 95% confidence intervals (CIs) reported the probability of a UB-related death. Deaths without cause specified were combined with non-UB related deaths for descriptive analysis and logistic regression modelling. Descriptive statistics characterised the following demographic variables for all dogs and compared variation between UB-related and non-UB related deaths: sex; purebred status; bodyweight; neuter status; age at death; KC breed grouping; and insurance status.

Binary logistic regression and multivariable model building were undertaken using the statistics package R (R Foundation for Statistical Computing, Vienna, Austria. URL: https://www.R-project.org/, accessed on 12 December 2020). Evaluation of an outcome of death from a UB was assessed against the following risk factors: bodyweight; age at death; sex; neuter status; purebred status; individual breed; and KC breed group. As in Boyd et al. [37], Labrador retrievers were chosen as the reference for individual breed analysis due to their reputation for being a friendly family dog, regular employment as service dogs, and popularity ensuring a substantial baseline breed against which to compare the others.

Risk factors with liberal associations (*p* < 0.2) were taken forward for multivariable evaluation. The variables “Purebred”, “KC breed group”, and “bodyweight” were not simultaneously considered in multivariable modelling but were instead used individually to replace the breed variable in the final multivariable model developed from “breed”, “age at death”, “sex”, and “neuter status”. The final multivariable mixed model was undertaken with the lme4 package [40], using the clinic code as a randomiser. Model development used manual backwards stepwise elimination. The effect of adding individual pairwise interactions to the full model was assessed. The χ² test compared categorical variables, whilst the quality of the model was assessed using the Akaike information criterion (AIC) and the deviance difference test. Statistical significance was set at *p* < 0.05.

## 3. Results

The sampling frame for this study comprised 157 veterinary clinics and 933,353 dogs. A recent count of veterinary clinics in Australia found a total of 2260 registered companion animal veterinary clinics [41], of which 6.9% were represented by this study. A total of 6221 deactivated EPRs were extracted within the specified timeframe, of which 4341 were manually confirmed as individual dogs that had died aged three years or under and thus included in the following analysis. There was an even distribution according to sex (50.2% male, 49.1% female), and 29.7% were neutered. Of these 4341 records, 1160 (26.7%) were deaths attributed to UB-related causes, 2746 (63.3%) were non-UB related deaths (including instances where humane euthanasia was elected when the owner could not afford treatment), and 435 (10.0%) records did not specify the cause of death. Deaths ascribed to UBs in dogs aged three years and under were estimated to have a prevalence of 29.7% (95% CI 25.4–28.1) among the 3906 records where cause of death was specified. Following the methodology in Boyd et al. [37] and assuming that the 5.1 million dogs in Australia [1] have an average lifespan of 12 years, there are an estimated 425,000 canine deaths per year. Of these, 9.6% (*n* = 40,800) are assumed to be among dogs aged under three years, and they project to a prevalence of 29.7%; meaning that an estimated 12,118 dogs in Australia die from a UB-related cause every year.

Mechanism of death was not recorded for 104 (2.4%) of the 4341 records. Of the remaining records, 3373 (79.6%) deaths were through euthanasia, and 864 (20.4%) were unassisted. Of the unassisted deaths: 207 (24.0%) were due to RTAs; 30 (3.5%) were due to a dog attack; and the remaining died due to non-UB related reasons. Only two dogs in the sample were noted as being insured, and therefore insurance status was excluded from statistical analysis. Both of these dogs had non-UB reasons ascribed as the cause of death.

Table 1 reports the descriptive statistics and binary logistic regression results for the following categorical risk factors: bodyweight; age at death; sex; and neuter status. Note that bodyweight information was available for only 767/4341 records, and the last value recorded did not always correlate with the date of the last EPR.

### 3.1. UB-Related Demographic Data

The median age at which a dog died due to an UB was 1.45 years (interquartile range (IQR): 0.82–2.03, range: 0.0– ≤ 3.0). The median age at which a UB was first recorded in the clinical notes was 0.9 years (IQR: 0.48–1.42, range: 0.0– ≤ 3.0). The median bodyweight recorded at death was 16.5 kg (IQR: 7.35–25.50, range: 0.9–70). Sex was recorded for 1159/1160 records, of which 526 (45.4%) dogs were female. Neuter status was recorded for all 1160 records, and 489 (42.2%) were recorded as neutered. There was a record of gonadectomy for 185 dogs, with the procedure occurring at a median age of 0.7 years (IQR: 0.47–0.82, range: 0.2–2.2). Of the 1160 dogs that died due to a UB-related cause, the clinical notes indicated that neutering was performed at least in part to address the UB for eight dogs, “neutering was discussed” with the owner in 77 (6.6%) records with no further details provided, and for two dogs neutering had been discussed in relation to an UB but the dogs died prior to being gonadectomised. Acquisition source was recorded in 147 (12.8%) records: 86 (58.5%) were adopted, 37 (25.2%) were sourced from a breeder, 9 (6.1%) were bred by the owner, 7 (4.8%) were acquired through online marketplaces, 5 (3.4%) from backyard breeders, and 3 (2.0%) from pet shops.

Table 2 reports descriptive statistics and binary logistic regression results for breed related categorical risk factors: purebred versus crossbred; individual purebred breeds, including the grouped “other purebreds” breed; and KC breed groups. Breed information was available for 1145/1160 (98.7%) dogs that had UB-related deaths, of which 537/1160 (46.3%) were purebred dogs. Of these purebred dogs, the most common individual breeds were the Staffordshire bull terrier (*n* = 69, 12.8%), Australian cattle dog (*n* = 40, 7.4%), American Staffordshire terrier (*n* = 40, 7.4%), border collie (*n* = 25, 4.7%), and German shepherd dog (*n* = 22, 4.1%). The most prolific KC breed group were the terriers, which made up 165/1160 (14.2%) of dogs with UB-related deaths.

### 3.2. Non-UB Related Demographic Data

Of the dogs that died due to causes other than UB (*n* = 3181), the median bodyweight was 11.65 kg (IQR: 4.6–24.3, range: 0.1–84.0 kg), and the median age at death was 0.71 years (IQR: 0.3–1.8, range: 0.0–3.0 years). One thousand six hundred and seventy-two (52.6%) dogs were purebred; 1498 (47.1%) were female and 801 (25.2%) were neutered. Of the 1672 purebred dogs that died of non-UB related causes, the most common breeds were: Staffordshire bull terriers (*n* = 161, 9.6%); German shepherd dogs (*n* = 107, 6.4%); Labrador retrievers (*n* = 92, 5.5%), Rottweilers (*n* = 85, 5.1%), and greyhounds (*n* = 84, 5.0%) (Table 2).

### 3.3. Types of Undesirable Behaviours Exhibited

Aggression, RTA, dog attack, and anxiety/nervousness were the most common UBs reported (Table 3). Most dogs dying from UBs had only one UB recorded (*n* = 900, 77.6%), 208 (17.9%) had two UBs; 34 (2.9%) dogs had three; 14 (1.2%) had four; three dogs (0.3%) had five; and only one dog (0.1%, a German shepherd dog) was noted to have six UBs. Notably, whilst some deaths had multiple contributory UBs ascribed, the majority of RTA (90.1%) and dog attack (92.9%) related deaths only had the single UB category ascribed as the contributory cause of death.

**Figure 1:**
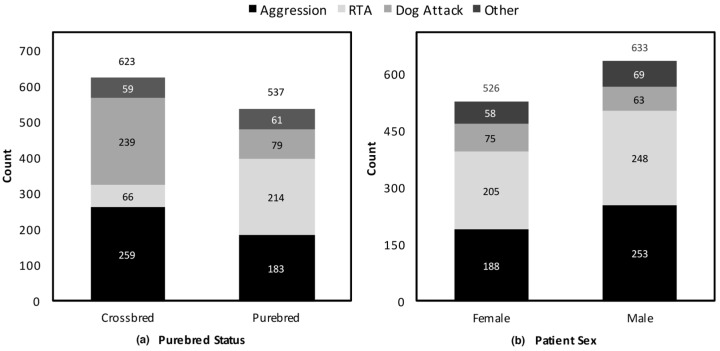
Prevalence of the three predominant UB disorder terms amongst (**a**) purebred and crossbred dogs, and (**b**) male and female dogs who died due to a UB-related cause: road traffic accidents (RTA); dog attack; and aggression. The fourth category (“other”) groups the remaining 13 UB disorder terms (Table 4) into one category.

Figure 1 shows the variance between UB categories as reported between purebred status and patient sex. Whilst prevalence of UB categories are comparable between sexes, crossbred dogs were overrepresented in the aggression and dog attack categories, and underrepresented in the RTA category.

**Figure 2:**
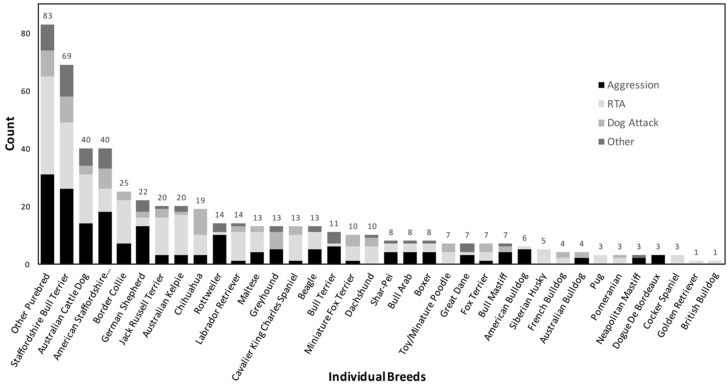
Proportion of the three predominant UB disorder terms that contributed to individual breeds of dogs dying due to UB-related causes: road traffic accidents (RTA); dog attack; aggression; and other, which grouped the remaining 13 UB disorder terms into one category. The total breakdown of individual breeds captured in this study sample can be found in Table 2.

Prevalence of UB categories between breeds are shown in Figure 2. Of the dogs that died due to UB, aggression was a contributing cause of death for 100% Dogue de Bordeaux (3/3), 92.9% Rottweilers (13/14), 83.3% American bulldogs (5/6), 81.8% bull terriers (9/11), 75% Australian cattle dogs (60/80), and 41.6% of crossbreds (259/623). The breeds that had RTA as the primary UB contributing to cause of death were Jack Russell terriers, Australian kelpies, Labrador retrievers, Cavalier King Charles spaniels, miniature fox terriers, and dachshunds. The breeds with RTA listed as the only UB contributing to cause of death were Siberian huskies, pugs, cocker spaniels, golden retrievers, and British bulldogs. The Chihuahua was the only breed reporting a majority representation of UB deaths due to a dog attack.

### 3.4. Treatment Prescribed for UB

No treatment was specified for 961/1160 (82.8%) dogs that died of UB. Six dogs were offered intervention by the clinician, but the owner declined. Two records indicated that ‘the owner had tried’ an intervention but provided no further clarification. Of the remaining records that specified an intervention to address the UB prior to euthanasia, the following proportions were found: at least one attempt at rehoming, including surrendering to a shelter, or placement into a foster home (*n*= 78, 23.6%); seeking advice from a trainer (*n* = 68, 20.5%); behaviourist referral (*n* = 65, 19.6%); pharmaceutical therapy (*n* = 68, 20.5%); advice from the attending veterinary clinician (*n* = 39, 11.8%); and neutering (*n* = 8, 1.5%). Note that an individual dog could have any combination of the aforementioned interventions applied, and the clinical notes did not always contain follow-up information regarding owner compliance or the success level of the interventions.

### 3.5. Statistical Method

Binary logistic regression modelling identified seven liberally significant (*p* < 0.20) risk factors that were then used for multivariable model building: bodyweight; age at death; patient sex; neuter status; purebred status; KC breed group; and individual breed (Table 1 and Table 2). Multivariable model building was developed using manual backward stepwise elimination, the full model contained the following terms:

Model 1: UB_death~Breed_analysis + Patient.Sex + Neutered + Age.Group

Further models were tested, replacing breed with: purebred status (Model 2); KC breed group (Model 3); and bodyweight (Model 4).

As all reduced models returned significantly reduced deviance using the deviance difference test and the full model returned the lowest AIC score, all reduced models were rejected and the full model was retained. Pairwise interactions were trialled, which resulted in the following analysis of variance (ANOVA) table, where the following terms were found to be significant:

Breed~Neuter Status (LR χ²: 49.37, df = 36, *p* = 0.068);

Breed~Age Group (LR χ²: 93.75, df = 72, *p* = 0.044);

Neuter Status~Age Group (LR χ²: 16.57, df = 2, *p* < 0.001).

Each pairwise interaction was added individually to the full model, where the following model performed the best compared to the full model alone:

UB_death~Breed_analysis + Patient.Sex + Neutered + Age.Group + Neutered: Age Group.

The validity of adding a second interaction term (breed/patient sex, breed/neuter status, breed/age, sex/neuter, and sex/age) was trialled and the model outcome assessed using the aforementioned methods but no significance was found. Therefore, this multivariable mixed model was retained, and the results can be seen in Table 4 and Table 5.

### 3.6. Results from Statistical Evalation

Among the breed associated variables, crossbred dogs had significantly increased odds (odds ratio (OR) 1.43, 95% CI: 1.24–1.65, *p* < 0.001) of dying due to an UB-related cause compared to purebred dogs. Of the KC breed groups, the following were found to be at decreased odds of death from a UB compared to non-KC recognised breeds (Table 4): hounds (OR = 0.54, 95% CI: 0.37–0.78, *p* < 0.001); non-sporting (OR 0.45, 95% CI: 0.32–0.65, *p* < 0.001); utility (OR 0.44, 95% CI: 0.32–0.60, *p* < 0.001); and gun dogs (OR 0.37, 95% CI: 0.24–0.56, *p* < 0.001). Dogs in the bodyweight category of 10.0–19.9 kg had 1.28× the odds (95% CI: 0.99–1.67) of death from UB compared to dogs in the bodyweight category of <10 kg.

Patient sex was not shown to significantly impact odds of a UB related death. Compared to dogs aged < 1.0 years, dogs in the 1.0– < 2.0 age category had 3.71× the odds (95% CI: 2.98–4.64, *p* < 0.001) of death due to UB, and dogs in the 2.0– ≤ 3.0 age category had 3.19× the odds (95% CI: 2.54–4.00, *p* < 0.001). Dogs that were neutered had 2.5× the odds (95% CI: 1.86–3.36, *p* < 0.001) of death due to UB. Neutered dogs in the < 1.0 year age category was shown to have higher odds at death due to a UB related cause than both the 1.0– < 2.0 age category (OR 0.44, 95% CI: 2.54–4.00, *p* < 0.001) and the 2.0– ≤ 3.0 age category (OR 0.49, 95% CI: 2.54–4.00, *p* < 0.001) (Table 5).

Sixteen individual breeds showed significantly higher odds of having a UB ascribed as to the cause of death compared to the Labrador retriever (Table 5). The breeds found to be at the highest odds (OR ≥ 3) were as follows: Australian cattle dogs (OR 4.77, 95% CI: 2.33–9.77, *p* < 0.001); American Staffordshire terriers (O: 4.69, 95% CI: 2.28–9.63, *p* < 0.001); miniature fox terriers (OR 4.08, 95% CI: 1.47–11.31, *p* = 0.007); bull terriers (OR 3.18, 95% CI: 1.47–11.31, *p* = 0.018); dachshunds (OR 3.17, 95% CI: 1.19–8.44, *p* = 0.021); shar-peis (OR 3.01, 95% CI: 1.05–8.58, *p* = 0.040); and the Cavalier King Charles spaniels (OR 3.0, 95% CI: 1.22–7.39, *p* = 0.017). Compared with the Labrador retriever, crossbred dogs were shown to have 2.86 × the odds of dying from a UB-related cause (95% CI: 1.59–5.13, *p* < 0.001), and the grouped other purebred variable showed 2.20× the odds (95% CI: 1.17–4.14, *p* = 0.015).

## 4. Discussion

For decades, UBs have been recognised as one of the most common causes of death in young dogs [32,42]. The current study found that 29.7% of dogs within the study sample that died at three years of age or under whilst attending primary care practices in Australia had an UB ascribed as a cause of death. This is slightly lower but comparable to the 33.7% prevalence reported by the companion study of UK dogs [37].

### 4.1. UB Demographic Data

Back in 1988, reporting on U.S. data, McKeown and Leuscher [42] estimated that one in four dogs and cats were either euthanased or relinquished within 12 months of acquisition. Unfortunately, that situation does not seem to have changed. Dogs that died within the 1.0– < 2.0 age category had the highest odds (OR 3.71) of death attributed to an UB when compared to dogs in the < 1.0 age category. Owners who acquire a small, cute puppy are sometimes ill prepared for the challenges that emerge when the puppy approaches adulthood. Sexual maturity is reached by six to nine months, while social maturity commences at approximately 12 months and is generally complete by 36 months of age [15]. During social maturity, significant changes in the brain manifest as changes in behaviour [17]. Indeed, there is recent evidence that dogs may go through a rise in conflict behaviour as they approach puberty [43]. It seems that while owners may be willing to excuse UBs in puppies, assuming that they will “grow out of it” or perhaps finding the behaviour more novel than undesirable, their tolerance of UBs is often finite. A dog’s behavioural responses, and especially those associated with aggression, are widely viewed as increasingly less plastic and more difficult to modify as the dog matures [17]. This leaves a narrow window of opportunity to provide guidance and help to owners of young dogs displaying UBs [31]. The current study showed only a six-month difference between the median age of first reported UB and age at euthanasia due to UB.

The median age of death due to a UB in the current study was 1.45 years, which is only 0.05 years lower than the equivalent age reported by the companion study in the UK [37]. However, the current study found that the median age at which case dogs first displayed an instance of UB prior to death was 0.9 years, which is six months earlier than that reported by Boyd et al. [37]. This suggests that Australian veterinarians may have an opportunity to assist owners of dogs exhibiting UBs that are younger, and possibly more malleable, than their UK counterparts. This is an opportunity, if pursued, that may save dogs’ lives and so merits close scrutiny.

Interestingly, whilst there was a higher proportion of female dogs dying of UB in Australia compared to the UK [37], sex had no significant impact on the odds of death due to a UB in the Australian cohort. This is not consistent with previous studies that showed males to be at increased risk of death due to UB [5,10,13,18,37]. That said, whilst female dogs had a lower prevalence of aggression, RTA, and other UBs, they had a higher prevalence of involvement in dog attacks than male dogs (Figure 1). This finding is consistent with data reported by other studies where more aggression problems occur in males than females, particularly intermale and stranger-directed aggression, whilst higher rates of interfamilial aggression have been reported in females [44]. It is normal for dogs from the same household to spend unsupervised time together and to share common resources in close proximity to each other, compared to time spent with unfamiliar dogs. Any conflict that arises, when there is no human around to intervene, has an increased risk of escalation to life-threatening injury. Further research to explore this difference could include: collecting data on domestic dog-keeping practices; the use of aversive stimuli in training [19]; establishing whether the dogs involved in interdog aggression belong to the same household or are unfamiliar; exploring differences between same-sex aggression and male–female aggression; and assessing the role of competition for resources.

The acquisition source of dogs was recorded in only 12.5% of dogs that died with UB related reasons, but had comparable proportions to the data reported by Boyd et al. [37]. This is surprising due to the disparity between purebred and crossbred status between the two study samples. One acquisition category in this study that was not captured by the UK study was online acquisition. However, this category contained only seven dogs (0.6%) in the current study. Previous research has shown the influence of acquisition source of a dog on levels of owner reported UB, with increased reports of unfriendliness, aggression, and excitability in dogs sourced from pet shops compared to those bred by the owner [21].

### 4.2. Variables with a Genetic Basis

Crossbred dogs (OR 1.43) were significantly more likely to die from UB related causes than purebred dogs (Table 4). They were proportionally more common in the current population than in the UK study [37]. This is likely to reflect the true proportion of crossbred dogs in Australia as their continually rising popularity is estimated to comprise up to 46% of Australia’s total companion dog population, more than any single distinct breed of dog [1]. Compared with crossbred dogs, purebred dogs in Australia are reported to have been more likely chosen for their temperament, they are less likely to have been desexed, and their owners have been found to be more likely to spend more money on their dogs and visit the veterinarian [1]. Bennett and Rohlf (2007) [21] postulated that the increased prevalence of UBs in crossbred compared to purebred dogs did not have a genetic basis, but rather reflected differences in the level of commitment between owner groups, and that owners who spend more on their dogs typically are more committed to their care and training [21].

The “Crossbred” category in the current study contained dogs of a mixed heritage and “designer breeds” (e.g., cavoodles). The popularity of these first generation crosses in Australia has been growing this decade [1]. Australian crossbred dogs have been identified as likely to comprise a mix of the popular purebred dogs, namely the kelpies, Maltese, Jack Russell terriers, Staffordshire bull terriers, and cattle dogs [1]. Aside from the Maltese, these breeds also happen to be those identified by this study as at risk of dying due to a UB related cause (Table 5). This may reflect the popularity of these breeds and the consequent prevalence of their crosses in Australia.

The breeds identified by the current study as being most at risk of dying due to UB (descending OR > 3, *p*-value < 0.05) were as follows: Australian cattle dog; American Staffordshire terrier; miniature fox terrier; bull terrier; dachshund; shar-pei; and the Cavalier King Charles Spaniel. This is contrary to the study conducted at an Australian behaviour referral clinic in Queensland where Australian cattle dogs were at a significantly lower risk of aggression compared to other breeds, perhaps highlighting the effect of a wide range of dog-keeping practices that can be employed across different contexts [13]. The Queensland study also used mixed breed dogs as their reference breed. The difference in breed popularity between the UK and Australia may explain the international differences in breeds found to be most at risk of dying due to a UB related cause, for example the Australian cattle dog in Australia, and the West Highland white terrier in the UK [37]. The current study found that American Staffordshire terriers had a much higher risk of death due to a UB related cause (OR 4.69) than Staffordshire bull terriers (OR 2.68), despite similar proportional data regarding types of UB causing death (Figure 2). This difference may further suggest that different dog keeping practices may influence behaviour, as both breeds are among the ten most popular breeds in Australia [1], and are morphologically similar in both height and cephalic index [14].

Risk of dying from a UB according to breed may also reflect the type of UB. A higher proportion of deaths due to RTA was seen in working breeds such as Australian kelpies, Labrador retrievers, and Australian cattle dogs. It may be that these dogs are at increased risk of RTA due to increased exposure to traffic and allowance of roaming behaviours. Terriers were originally bred to chase, so if this behaviour is transferred from prey to motor vehicles, it follows that they are exposed to increased risk of RTA. The location of the dogs may also play a part, as fencing will vary between suburban and rural locations.

Greyhounds were predisposed to aggression-related behaviours and dog attack among the UB categories for the cause of death. The clinical notes indicated that this was likely due to a high proportion dogs being kept as racers. This could mean that owner tolerance levels for interdog aggression was low as even non-life threatening dog attack injuries (so-called marring) risks ending a racing dog’s career, or that the dogs were frequently exposed to high arousal states whilst kept in groups, which inherently increases the risk of interdog conflict. It is important to note that this study was not designed to differentiate between predatory aggression and other types of aggression. Breed specific legislation also exists within several Australian states that stipulate that all pet and racing greyhounds are to be leashed whilst in public, which may explain the noticeable lack of greyhounds in the RTA category.

The current KC breed group analysis affords a broader examination of breeds that may be at risk of death due to UBs that may not be captured through individual breed analysis due to variations in popularity and availability of certain dog breeds. The breed group forms the larger umbrella under which the breed phenotype allocates both appearance and behaviour, and both qualities are reflected to an extent in the relevant breed standards. The terrier breed group showed the highest prevalence of dogs that had UB related deaths. Although the increased OR was not found to be significant in the current study, Boyd et al. [37] reported similar results that were significant. Terriers may have a genetically based tendency to be less responsive to anthropogenic stimuli, a trait that may have been a by-product of selecting for dogs that had the ability to work independently in their traditional hunting roles [45]. This may make them more difficult to manage when highly aroused.

The AKC breed groups at lowest risk of dying from UB were the gun dogs and utility dogs. These were also the AKC breed groups that contained the smallest number of individuals. It is difficult to categorise dogs belonging to the utility group because it is characterised chiefly by breeds that do not fit into the other groups. Views may vary on what does and does not constitute an UB among owners of disparate breed groups. For example, aggression towards strangers at the house can be highly desirable in a guarding breed, and therefore never reported to a primary care practitioner. The working dog group had decreased odds (OR 0.83) of having a UB ascribed as the cause of death compared to non-AKC dogs (Table 4), despite containing dog breeds that are classically considered inappropriate for suburban life due to high drive and energy levels [46]. However, working dogs that are in work usually receive high levels of training. Whilst there may be a basis for attributing UB in working dogs to frustration arising from inadequate mental and physical stimulation, the current results encourage a systematic and individualised approach when investigating UB in dogs as many can live comfortably outside of their breeds’ historical occupations.

Bodyweight is a phenotypic metric that varies predictably with breed [9]. Dogs in the 10–19.9 kg weight category were found at the highest risk of a UB-related death (liberally significant at *p*-value = 0.062). Dogs in the terrier breed group generally reach an expected bodyweight of 6–15 kg. In the current study, the prevalence of dogs dying due to a UB related cause decreased as the bodyweight rose (Table 1), a trend reflective of previous studies that aligned breeds with certain behaviours [9,37]. Owners of larger breeds may have less tolerance of UBs as they recognise the damage that a relatively large dog can inflict. In a survey of self-reporting dog bite victims, 70% of bites were by dogs considered medium or large [47]. As a consequence, breeders of large breeds may practice routine training and socialisation of their dogs and select for temperament more than breeders of smaller breeds, and select for a similar awareness among those who may obtain puppies from them as part of optimising public perception of the breed.

Small dogs are more likely than large dogs to show UB [9], possibly because their owners tolerate UBs for longer and fail to manage them appropriately. Owners of small dogs may also be more likely to underestimate the amount of exercise a small dog may need, and therefore allow the dog to accrue higher levels of frustration from unspent energy that manifests in hyperexcitability and anxiety-related UBs.

### 4.3. The Effect of Neutering

The current study showed that compared to entire dogs, neutered dogs had increased odds (OR 2.5) of an UB being ascribed as a cause of death. There are several possible explanations for this. Owners of intact animals may be, or may consider themselves to be, more experienced and able to manage the suite of behaviours exhibited by an entire dog, and are therefore also able to better manage other UBs. The increased level of care provided by owners who bring their animals to be surgically neutered compared to those who do not may come with a higher level of expectation regarding acceptable standards of behaviour [48]. Additionally, some of the dogs that are euthanased as a result of UB may have had neutering alone administered in a weak attempt to resolve the UB, rather than comprehensive behavioural therapy.

Only 29.7% of the total 4341 dogs captured by this study were neutered. This is much lower than the 81% quoted in the most recent Australian pet survey [1]. The proportion of neutered dogs that died due to UB was found to be 42.2%. The UK study [37] reported 76.5% of dogs whose death was attributed to UB were neutered, but neuter status was recorded in only one third of dogs within that study. Relatively low lifetime exposure to gonadal hormones has been shown to increase the frequency of aggression [49] and anxiety-related behaviours, including territorialism, fear, and reactivity to visitors to the household [18].

Preliminary investigations have shown that neutering used as a management method to decrease UBs achieved improvements in less than a third of aggression-related behaviour cases, and fewer than half of owners reported more than 90% improvement on roaming (which is linked to increased incidence of RTAs) [50]. The current analysis showed that neutered dogs under one year of age had significantly higher odds of dying due to an UB related cause than dogs in the 1.0– < 2.0 and 2.0– ≤ 3.0 age categories (see Table 5, ‘Combined’). Early age desexing in male dogs has been associated with increased risk of aggression towards family members [49] and, in both sexes, increasing signs of separation anxiety-related disorders [10]. However, there is some evidence that the younger the dog is neutered, the lower the reported incidence of the dog exhibiting escape behaviours, which may influence the prevalence of deaths due to RTA [49].

Whilst the current study was not designed to follow the dogs that had been neutered as a response to a UB and subsequently survived, the association it has revealed between death due to UB and neuter status has been observed by others [10,18]. Dogs neutered for UBs are more likely to have required prior treatment for those UBs [51], and, in most cases, neutering has not been shown to increase trainability [52]. Even if neutered animals were aggressive prior to neutering, the higher incidence of aggression in the current study, despite the sizeable proportion of neutered dogs in the sample, indicates that the procedure is not therapeutic for management of UB. This presents a paradox in which neutering on a population level decreases the number of unwanted dogs through indiscriminate breeding, yet may increase the likelihood of individual dogs exhibiting UBs, making them less desirable pets, which renders them just as unwanted [10].

### 4.4. Types of UB and Interventions Offered

Aggression was the most common UB category related to death in dogs of three years of age or under. This is consistent with reports from behavioural clinics [13], shelters [36], and owner surveys [31]. The threat of injury is often the most pressing concern for owners of aggressive dogs, and a repeat episode of aggressive behaviour is often the trigger for owners to euthanase the dog. Aggression and disobedience consistently feature among the top risk factors for owner dissatisfaction and subsequent abandonment or euthanasia [13,49,53]. Whilst most UB categories are distinct, they are strongly correlated and comorbidities are common [54], for example interpet and interfamily conflict types of UB may also manifest as canine aggression. Dog attacks have an actively aggressive component, for example in disputes over resources, but like many aggressive problems exhibited by dogs they can also be motivated by anxiety, fears, and/or phobias, rather than unprovoked aggression [5]. Aggression associated with concurrent chronic disease and, most notably, chronic skin disease was also noted in the EPRs in the current study. Dogs with skin allergies have been previously associated with increased problem behaviours and lower trainability scores [55]. A highly aroused dog can also be motivated to aggress if it enters a negative emotional state [56].

Once a dog exceeds an emotional threshold, its owner may misinterpret its overstimulation as disobedience, and incorrectly assign blame for the motivation for the UB. Escape behaviour, destructiveness, and excessive vocalisation may be manifestations of frustration in dogs with unmet behavioural needs [13], but may also arise in dogs with separation-related distress [28]. Breed and limited time spent at home by the owner often influence the expression of these UB categories [13]. Additionally, there may be causal interactions between an animal’s behaviour and its owner’s personality and level of attachment [2], some of which predispose to inadequate socialisation and a tendency towards aggressive assertiveness towards their dog [57]. The importance of both learning and genetic influences on behaviour cannot be overstated [18], as reflected in both owner breed selection and subsequent steps taken to ensure adequate socialisation after acquisition of a new dog. Adequate socialisation is pivotal in ensuring a strong, positive human–animal relationship that is ultimately crucial for many companion animals’ survival [58].

The proportion of mortality due to RTA was similar in both the UK (39.0%) [37] and Australia (41.4%). Death due to RTAs was classified as UB mortalities, as RTAs may result from dogs showing poor recall, limited traffic training, and escape behaviours, which are all associated with UBs [46]. The current overall high incidence of death due to RTA (Table 3) has implications for better regulations of dog keeping, such as mandatory leashing of dogs when crossing thoroughfares with high traffic flow.

There were more UB-related deaths attributed to a dog attack in the Australian study (16.8%) than in the UK study (5.9%) [37], although the proportion of unassisted deaths due to a dog attack were identical (3.5%). It is not possible to state whether this reflects a difference in dog socialisation practices, different rates of euthanasia, or severity of injuries in each case requiring attention at a primary care practice. The authors acknowledge that the current study could not identify the other dog(s) involved in the recorded dog attacks, if it was more than 3 years of age, or whether it died from the attack. The increased prevalence of death due to RTA and a dog attack in Australia could be due to favourable weather patterns encouraging owners to exercise their dog outside, increasing exposure to road traffic and other dogs, whilst inclement weather in the UK predisposes companion dogs to less outdoor activity [59]. Similarly, purebred dogs in the current study were overrepresented in deaths due to RTA compared to crossbreds (Figure 1), possibly as a reflection of owners of purebred dogs being more likely to take their dog to public places, such as the beach or park [1].

In the current study, an overwhelming majority of dogs that died due to UB(s) either did not have an intervention recorded in the clinical notes, or none was offered. The most popular interventions represent those that demand the least effort (surrendering the animal) as opposed to those requiring most time and money (pharmaceutical therapy and behavioural referral). It is encouraging to note that, proportionally, more behavioural and pharmacological interventions were provided to the current cohort than were reported by Boyd et al. [37] five years ago. However, there is still scope for considerable improvement. For example, dogs recorded as difficult to handle were frequently reported in the clinical notes as being muzzled but rarely sedated. There is a high risk that the dog may learn from this negative experience to become more reactive at the next veterinary visit, especially without the liberal use of food rewards. The role of the veterinarian at the forefront of animal care is undermined if addressing behaviour is not considered an integral aspect of animal welfare [60].

Whilst it is worth noting that these results suggest a higher proportion of overall deaths through euthanasia in Australia (79.6%) than in the UK (76.2%) [37], no data are available to explain the reasons for this difference. Areas of further investigation could include the effect of cultural attitudes to euthanasia, how often euthanasia was chosen due to financial restraints, or how many owners elected the low-cost option of taking their dog to a shelter for euthanasia. UBs are a common cause of relinquishment to shelters in both countries [31,61]. While Australia has a lower estimated dog population than the UK [1,32], RSPCA Australia reported an intake of 33,863 dogs in the 2018–2019 financial year [30] whereas RSPCA UK reported an intake of 10,564 dogs in 2019 [62]. The higher euthanasia rates in Australia may also reflect factors that increase the frequency of relinquishment, including tolerance of UB, availability of treatment options (such as access to behavioural referral), variance in the average size of the dog or the ways dogs are kept.

### 4.5. Limitations

For several reasons, the current study is likely to have underestimated the national scale of canine euthanasia due to UB. For example, it does not include data from the considerable number of animals surrendered to and subsequently euthanased at animal shelters due to UBs [36], or the proportion of animals that are lost or abandoned for reasons related to UBs but never presented to a primary care veterinary clinic. Ten per cent of dogs aged three years or under in this study were confirmed as having been euthanased in the clinical notes without any further explanation as to why the procedure was being performed. Whilst this somewhat detached method of recording death of a companion animal may simply reflect a lack of detail in the clinical records, it may also represent a severed owner–animal bond resulting from a UB.

It is possible that the breed analysis may have been affected by some misclassification of dog breeds when the EPRs were being completed at their respective veterinary practices. Breed and/or pedigree certification is rarely required in order to register an animal as purebred within a veterinary clinic database. Consequently, there is no guarantee that misclassification of dog breed did not occur, be it a purebred dog that has been recorded as another morphologically similar but divergent breed, misclassified as a crossbred, or vice versa. Furthermore, this study did not define crossbred dogs as mixed-breeds because the necessary information was not available to distinguish mixed-breed from true first-generation crosses. This methodology may have diluted the individual breed analysis and affected the prevalence of breed-specific traits discussed. However, the primary aim of the study was to produce a comparable analysis to Boyd et al. [37], so the authors were obliged to follow suit even though more granularity in the demographic may have been preferred. Again, following the methodology of Boyd et al. [37], the current study relied on one researcher to manually open records to confirm cases as individual dogs.

Some dog breeds recorded in the Australian EPR did not fall within the breeds as outlined in Boyd et al. [37], and were categorised as crossbred in the purebred/crossbred analysis. This also subsequently altered the KC breed groupings. The breed categorisation guide used by this study can be viewed in File S2.

Bodyweight categories were assigned by recording the last available weight for each dog through evaluation of EPR. However, this value may not always correlate with the final adult weight range of the animal. Dogs that were significantly underweight, obese, or their weight inconsistently recorded may have been inadvertently assigned to an upper or lower weight category.

Finally, whilst the current methodology aimed to replicate that of Boyd et al. [37], there were two points at which it deviated. Firstly, Boyd et al. [37] surveyed clinical notes, which spanned a five-year period, whereas data for the current study were only available for just over a four-and-a-half-year period. This temporal shortfall may have been offset by the larger sample population of confirmed patient deaths in this study, 4341 compared to 1574 [37]. Secondly, data included in Boyd et al.’s [37] univariable logistic regression included all three categories of dogs that were confirmed as deceased at three years of age or younger (*n* = 1574): UB related; non-UB related; and reason for death not specified. The final multivariable logistic regression analysis excluded dogs from the final category where the cause of death was not specified. In contrast, the current study performed both the binary and final multivariable analysis on all three categories.

### 4.6. Implications for the Future

In keeping with a One Welfare approach [63], preventative action ultimately seeks to improve not only canine welfare but also the welfare of any involved humans. Identifying risk factors helps to prevent problems from occurring. This is desirable not only because it means less harm comes to the dog and those around it, but behavioural modification takes time and often requires the owners to make lifestyle changes, which can be difficult to implement and sustain [64]. Preventative action can be taken on every level. Breeders who base selection of breeding stock on behavioural and physical traits should be encouraged. Socialisation of dogs may begin much earlier than the socialisation window reported to commence at 3 weeks of age so arguably, best practice should begin prenatally by ensuring a stress-free birthing environment for the bitch, and, following birth, should involve provision of regular gentling of the newborns [65]. Previous studies have demonstrated the effectiveness of giving veterinary behaviourists’ advice to puppy owners as a means of reducing UB [66]. Liberal employment of services such as doggy daycare and dog walkers can normalise dog behaviour and advance dog welfare when the urban dog owner spends most of their time away from home.

Previous surveys have identified that veterinarians fail to adequately address UB concerns raised by owners [67], which may be reflected in their lack of detailed clinical notes addressing routine behaviour. From the perspective of a veterinary practice, the time spent on behavioural consultations do not currently reap a proportional amount of revenue [68]. However, veterinary practices are appearing to place increasing emphasis on behaviour, with accreditation programs for minimal stress handling gaining in popularity. Many practices now run puppy behaviour classes or have a close association with a reward-based trainer. There is an increasing awareness that veterinarians should be a trusted resource for assistance with behaviour problems. This requires that veterinary clinical staff feel confident to deal with UBs and are allocated the time needed to thoroughly address the problem. Ensuring graduate veterinarians acquire a basic knowledge of behaviour and learning theory will aid in the prevention of stress and subsequent escalation of UBs in dogs for multiple scenarios [69]. The latter is even more pertinent now that UBs in dogs have been shown as a covert way of exhibiting pain [70,71], further supporting the need for detailed scrutiny of UB reports on a case-by-case basis. Indeed, such studies may help to clarify some of the causal links that are absent from the current report.

## 5. Conclusions

UBs are among the primary reasons for surrender of a dog to a shelter, but increasing evidence shows that it may also be the primary reason for euthanasia in young dogs. This study found that amongst dogs that died under three years of age, those aged between one and two years had the highest odds of dying due to a cause ascribed to a UB. Crossbred dogs were at higher risk of a UB-related death compared to purebred dogs, and neutered dogs were at higher risk compared to entire dogs. Increasing bodyweight was negatively correlated with the prevalence of a UB-related death. Aggression followed by RTA was the two main UB categories. Aggression was a contributing cause of death for 100% Dogue de Bordeaux, 92.9% Rottweilers, 83.3% American bulldogs, 81.8% bull terriers, 75% Australian cattle dogs, and 41.6% of crossbreds. The most common breeds that died due to RTA were Jack Russell terriers, Australian kelpies, Labrador retrievers, Cavalier King Charles spaniels, miniature fox terriers, and dachshunds.

There was only a six-month gap between the median age at which the UB is first mentioned in the EPR and the age at euthanasia due to UB. This small window of opportunity for behavioural therapy emphasises the importance of preventative action, and the application of thorough, context specific treatment by the veterinary professional when treatment is sought. No intervention was indicated in the clinical records for most dogs in this study that had UB-related deaths.

These results confirm the significant impact of UBs affecting the welfare of dogs in Australia, and highlights the impact different dog keeping styles may have on the expression of UBs. It offers opportunities to design better education of dog behaviour for owners and veterinary professionals, and to help guide further research into the modification and management of UBs.

## Figures and Tables

**Table 1:** Descriptive and binary logistic regression results for risk factor associations (bodyweight, age at death, sex, neuter status, and insurance status) with UB and non-UB related deaths amongst dogs attending primary-care veterinary practices in Australia that died at three years of age or under (*n* = 4341).

Variable	Category	UB Number (%)	Non-UB Number (%)	Odds Ratio	95% CI	*p*-Value
Bodyweight (kg)	<10.0	196 (16.9)	797 (25.1)	Base		
	10.0–19.9	144 (12.4)	356 (11.2)	1.64	1.28–2.11	<0.001
	20.0–29.9	147 (12.7)	341 (10.7)	1.75	1.37–2.25	<0.001
	30.0–39.9	60 (5.2)	169 (5.3)	1.44	1.03–2.01	0.031
	≥40.0	38 (3.3)	81 (2.5)	1.91	1.25–2.87	0.002
	Not recorded	575 (49.6)	1437 (45.2)	1.63	1.36–1.96	<0.001
Age at death (years)	<1.0	365 (31.5)	1853 (58.3)	Base		
	1.0– < 2.0	390 (33.6)	610 (19.2)	3.25	2.74–3.85	<0.001
	2.0– ≤ 3.0	405 (34.9)	718 (22.6)	2.86	2.43–3.38	<0.001
Sex	Female	526 (45.3)	1654 (52.0)	Base		
	Male	633 (54.6)	1498 (47.1)	1.09	0.95–1.25	0.212
	Not recorded	1 (0.1)	29 (0.9)	0.10	0.01–0.46	0.023
Neuter Status	Entire	671 (57.8)	2380 (74.8)	Base		
	Neutered	489 (42.2)	801 (25.2)	2.17	1.88–2.49	<0.001

**Table 2:** Descriptive and binary logistic regression results for risk factor associations (purebred status, breed, and Kennel Club (KC) breed group) with deaths that were or were not ascribed to undesirable behaviour (UB) amongst dogs attending primary-care veterinary practices in Australia that died before three years of age or under (*n* = 4341). Results have been ordered by descending odds ratio (OR).

Variable	Category	UB Number (%)	Non-UB Number (%)	OR	95% CI	*p*-Value
Purebred Status	Purebred	537 (46.3)	1672 (52.6)	Base		
	Crossbred	623 (53.7)	1509 (47.4)	1.29	1.12–1.47	<0.001
KC Breed Group	Not KC-registered	646 (55.7)	1560 (49.0)	Base		
	Terriers	165 (14.2)	339 (10.7)	1.18	0.95–1.44	0.127
	Working	119 (10.3)	307 (9.7)	0.94	0.74–1.18	0.574
	Toys	60 (5.2)	171 (5.4)	0.85	0.62–1.15	0.292
	Hounds	45 (3.9)	153 (4.8)	0.71	0.50–0.99	0.052
	Non Sporting	54 (4.7)	268 (8.4)	0.49	0.35–0.66	<0.001
	Utility	43 (3.7)	223 (7.0)	0.47	0.27–0.63	<0.001
	Gun Dogs	28 (2.4)	160 (5.0)	0.42	0.27–0.63	<0.001
Breed	Labrador retriever	14 (1.2)	92 (2.9)	Base		
	American Staffordshire terrier	40 (3.4)	58 (1.8)	4.53	2.32–9.31	<0.001
	Miniature fox terrier	10 (0.9)	15 (0.5)	4.38	1.63–11.72	0.003
	Australian cattle dog	40 (3.4)	62 (1.9)	4.24	2.17–8.68	<0.001
	Beagle	13 (1.1)	26 (0.8)	3.29	1.37–7.92	0.007
	Bull terrier	11 (0.9)	22 (0.7)	3.29	1.30–8.25	0.011
	Maltese	13 (1.1)	26 (0.8)	3.29	1.37–7.92	0.007
	Fox terrier	7 (0.6)	15 (0.5)	3.07	1.02–8.73	0.038
	Jack Russell terrier	20 (1.7)	43 (1.4)	3.06	1.42–6.74	0.005
	Cavalier King Charles spaniel	13 (1.1)	28 (0.9)	3.05	1.28–7.30	0.012
	Dachshund	10 (0.9)	22 (0.7)	2.99	1.15–7.61	0.022
	Staffordshire bull terrier	69 (5.9)	161 (5.1)	2.82	1.54–5.47	0.001
	Shar-pei	8 (0.7)	19 (0.6)	2.77	0.99–7.45	0.046
	Crossbred	623 (53.7)	1509 (47.4)	2.71	1.59–5.01	0.001
	Australian kelpie	20 (1.7)	50 (1.6)	2.63	1.23–5.75	0.013
	Chihuahua	19 (1.6)	48 (1.5)	2.60	1.21–5.73	0.015
	Border collie	25 (2.2)	64 (2.0)	2.57	1.26–5.43	0.011
	Other purebred	83 (7.2)	227 (7.1)	2.40	1.33–4.61	0.005
	Boxer	8 (0.7)	27 (0.8)	1.95	0.71–5.06	0.178
	Great Dane	7 (0.6)	24 (0.8)	1.92	0.66–5.17	0.208
	Bull mastiff	7 (0.6)	25 (0.8)	1.84	0.64–4.94	0.236
	Bull Arab	8 (0.7)	34 (1.1)	1.55	0.57–3.95	0.370
	Neapolitan mastiff	3 (0.3)	13 (0.4)	1.52	0.32–5.47	0.553
	Cocker spaniel	3 (0.3)	14 (0.4)	1.41	0.30–5.02	0.624
	German shepherd dog	22 (1.9)	107 (3.4)	1.35	0.66–2.85	0.416
	Toy/miniature poodle	7 (0.6)	35 (1.1)	1.31	0.47–3.44	0.587
	Siberian husky	5 (0.4)	26 (0.8)	1.26	0.38–3.65	0.679
	American bulldog	6 (0.5)	32 (1.0)	1.23	0.41–3.36	0.693
	Rottweiler	14 (1.2)	85 (2.7)	1.08	0.48–2.42	0.846
	Greyhound	13 (1.1)	84 (2.6)	1.02	0.45–2.30	0.967
	Australian bulldog	4 (0.3)	28 (0.9)	0.94	0.25–2.87	0.917
	Pomeranian	3 (0.3)	23 (0.7)	0.86	0.19–2.90	0.820
	Dogue de Bordeaux	3 (0.3)	25 (0.8)	0.79	0.17–2.65	0.725
	French bulldog	4 (0.3)	38 (1.2)	0.69	0.19–2.07	0.538
	Pug	3 (0.3)	30 (0.9)	0.66	0.14–2.18	0.531
	British bulldog	1 (0.1)	17 (0.5)	0.39	0.02–2.13	0.374
	Golden retriever	1 (0.1)	27 (0.8)	0.24	0.01–1.30	0.181

**Table 3:** Prevalence of the most common groups of undesirable behaviours (UBs) recorded as contributing to deaths from a UB among dogs attending primary-care veterinary practices in Australia that died at three years of age or under with an ascribed cause (*n* = 1160).

Undesirable Behaviour	*N* (%)	95% CI
Aggression	609 (52.5)	49.9–55.8
Road Traffic Accident (RTA)	480 (41.4)	38.0–43.8
Dog Attack	195 (16.8)	14.7–19.1
Anxious/Nervous	135 (11.6)	9.8–13.5
Inter-pet conflict	90 (7.8)	6.3–9.5
Limited examination	42 (3.6)	2.6–4.9
Excessive vocalisation	30 (2.6)	1.8–3.7
Inter-family conflict	27 (2.3)	1.5–3.3
Destructive	17 (1.5)	0.9–2.3
Hyper-excitability	12 (1.0)	0.5–1.7
Inappropriate elimination	9 (0.9)	0.4–1.5
Owner can’t cope	7 (0.6)	0.2–1.2
Limited training	2 (0.2)	0.0–0.6
Hyper-sexuality	1 (0.1)	0.0–0.5
Other behaviours	96 (8.3)	6.8–1.0

Note that some deaths had multiple contributory UBs ascribed.

**Table 4:** Final multivariate mixed model results for purebred, Kennel Club (KC) breed group, and bodyweight as risk factors associated with deaths ascribed to UBs amongst dogs attending primary-care veterinary practices in Australia that died at or before three years of age.

Variable	Category	Odds Ratio	95% CI	*p*-Value
Purebred Status	Purebred	Base		
	Crossbred	1.43	1.24–1.65	<0.001
KC Breed Group	Not KC-registered	Base		
	Terriers	1.07	0.86–1.34	0.540
	Working	0.83	0.65–1.06	0.128
	Toys	0.77	0.56–1.06	0.108
	Hounds	0.54	0.37–0.78	0.001
	Non Sporting	0.45	0.32–0.65	<0.001
	Utility	0.44	0.32–0.60	<0.001
	Gun Dogs	0.37	0.24–0.56	<0.001
Bodyweight (kg)	<10.0	Base		
	10.0–19.9	1.28	0.99–1.67	0.062
	20.0–29.9	1.01	0.77–1.31	0.950
	30.0–39.9	0.79	0.56–1.13	0.197
	≥40.0	0.9	0.58–1.39	0.639
	Not recorded	1.59	1.30–1.93	<0.001

**Table 5:** Final multivariate mixed model results for risk factors associated with deaths ascribed to undesirable behaviour (UB) among dogs attending primary-care veterinary practices in Australia that died at three years of age or under. The breed variable has been ordered by descending odds ratio (OR). Age group 1: < 1.0 years; age group 2: 1.0– < 2.0 years; age group 3: aged 2.0– ≤ 3.0 years.

Variable	Category	OR	95% CI	*p*-Value
Sex	Female	Base		
	Male	1.05	0.91–1.22	0.466
Age at death (years)	<1.0	Base		
	1.0– < 2.0	3.71	2.98–4.64	<0.001
	2.0– ≤ 3.0	3.19	2.54–4.00	<0.001
Neuter Status	Entire	Base		
	Neutered	2.5	1.86–3.36	<0.001
Combined	Neutered/Age < 1.0	Base		
	Neutered/Age1.0– < 2.0	0.44	0.30–0.65	<0.001
	Neutered/Age2.0– ≤ 3.0	0.49	0.34–0.73	<0.001
Breed	Labrador retreiver	Base		
	Australian cattle dog	4.77	2.33–9.77	<0.001
	American Staffordshire terrier	4.69	2.28–9.63	<0.001
	Miniature fox terrier	4.08	1.47–11.31	0.007
	Bull terrier	3.18	1.22–8.28	0.018
	Dachshund	3.17	1.19–8.44	0.021
	Shar-pei	3.01	1.05–8.58	0.040
	Cavalier King Charles spaniel	3.00	1.22–7.39	0.017
	Crossbred	2.86	1.59–5.13	<0.001
	Maltese	2.83	1.15–6.98	0.024
	Jack Russell terrier	2.78	1.25–6.17	0.012
	Fox terrier	2.71	0.90–8.17	0.076
	Staffordshire bull terrier	2.68	1.40–5.12	0.003
	Beagle	2.65	1.06–6.63	0.038
	Chihuahua	2.64	1.18–5.87	0.018
	Border collie	2.56	1.20–5.43	0.015
	Australian kelpie	2.30	1.04–5.08	0.039
	Other purebred	2.20	1.17–4.14	0.015
	Boxer	1.90	0.70–5.21	0.210
	Bull mastiff	1.72	0.61–4.90	0.307
	Bull Arab	1.68	0.62–4.51	0.306
	Great Dane	1.39	0.49–3.94	0.532
	Neapolitan mastiff	1.35	0.33–5.53	0.677
	Toy/minature poodle	1.30	0.47–3.61	0.611
	Rottweiler	1.20	0.53–2.73	0.663
	Siberian husky	1.19	0.38–3.75	0.766
	Australian bulldog	1.16	0.34–3.98	0.810
	American bulldog	1.14	0.39–3.31	0.809
	German shepherd	1.10	0.52–2.31	0.811
	Cocker spaniel	0.98	0.24–3.96	0.976
	French bulldog	0.79	0.24–2.63	0.699
	Pomeranian	0.76	0.20–2.93	0.686
	Pug	0.75	0.20–2.89	0.680
	Dogue de Bordeaux	0.72	0.18–2.78	0.630
	Greyhound	0.69	0.30–1.60	0.387
	British bulldog	0.31	0.04–2.59	0.280
	Golden retriever	0.23	0.03–1.86	0.168

## Data Availability

The data presented in this study are available on request from the corresponding author. The data are not publicly available due to conditions of ethical approval.

## Supplementary Materials

The following are available online at https://www.mdpi.com/2076-2615/11/2/493/s1; File S1: Appendix for Mortality resulting from undesirable behaviours in dogs aged under three years attending primary-care veterinary practices in the UK; File S2: VetCompass Breed groupings.
