# Supplementary material for: Mortality Resulting from Undesirable Behaviours in Dogs Aged Three Years and under Attending Primary-Care Veterinary Practices in Australia
Source: Animals (Basel). 2021 Feb 13;11(2):493. doi: 10.3390/ani11020493 (PMC7918417; doi:10.3390/ani11020493)
Supplement: Supplementary file 1 [file animals-11-00493-s001.zip › animals-1055081-supplementary/File S1.pdf]

Appendix for Mortality resulting from undesirable behaviours in dogs aged under three years attending primary-care veterinary practices in the UK

Appendix

Appendix Table 1: Definitions of behaviour at the general level: adapted from (Bamberger and Houpt, 2006; Lund et al., 1996)

Behaviour	Definition
Aggressive	Behaviour that includes growling, snapping, barking aggressively, biting, snarling and lunging. This could be directed towards: animals, children, inanimate objects, owners and people. It also includes resource guarding, territorial aggression, so-called dominant behaviour, dog-fights and nervous aggression.
Anxious/Nervous	Anxious behaviour, such as cowering, urinating, vomiting and shaking. This also include separation anxiety when the dog exhibits excessive anxious behaviour, following physical or visual separation from its owners, travel anxiety when the dog exhibits anxious behaviour when it is in a mode of transport and anxiety due to noise phobias.
Destructive	When the dog chews up or breaks items that the owner does not desire them to. Excluding when it is stated that the dog is destructive due to separation anxiety
Dog Attack	When the record states that the case dog was attacked by another dog. This includes being just a single dog bite but excludes bites that happened during play.
Excessive vocalisation	When the record states that the dog was barking, crying or whining excessively, this does not include if the dog is vocal for a short time (i.e. one night) due to a medical reason, or

	when it is stated that the dog is vocalising due to separation anxiety.
Hyper-excitability	When the dog is boisterous and the owner and/or vet are unable to control the dog.
Hyper-sexuality	Sexual behaviour, such as mounting, that is felt to be inappropriate and the owner and/or vet does not desire.
Inappropriate elimination	Elimination due to behavioural reasons in an undesirable location, excluding medical problems and dogs that have not yet been housetrained up to the age of 12 months.
Inter-family conflict	Conflict between the dog and humans in the household. The conflict could be in either direction, can be led by the case dog or directed towards the case dog.
Inter-pet conflict	Conflict between the dog and other animals in the household. The conflict could be in either direction, can be led by the case dog or directed towards the case dog.
Limited training	This is when the dog exhibits the behaviours: a lack of recall, pulling on the lead or when the record states that there are 'problems with training'.
Other behaviours	This includes the undesirable behaviours: herding, escaping from the house, car or garden, corprophagia, geophagia, locomotive stereotypes, neurological or physical changes (such as a change in gait, activity levels, the ability to recognise people etc.) and self-trauma.
Owner can't cope	When the record states that the owner is unable to cope with the dog. This could include owners that struggle with puppies.
RTA	When the dog is hit by a wheeled mode of transport, for example a bus, push bike, car, train etc.

Unidentified Behaviour	When the record states that the dog's behaviour has changed in a negative way or when the record states that the dog has 'behavioural problems'.
Limited examination	If the veterinary record stated that a full examination could not take place without the dog having to be sedated, muzzled or by using severe physical restraint, or a full examination cannot take place.

Appendix Table 2: Definitions of data extraction questions

Question	Answer	Definition
Was pharmacological therapy tried?	Pharmacological therapy tried	Pharmacological, pheromone and nutraceutical therapy including both systemic and topical treatments that have indications stating that they can benefit dogs with undesirable behaviours.
	No pharmacological therapy tried	
Was neutering due to behaviour?	Neutered due to behaviour	In the EPR, the veterinarian and/or owner states that the neutering decision related at least in part to behavioural reasons; including to prevent a behaviour or to help manage an existing behaviour.
	Neutered not for behaviour	There is evidence that the dog was neutered during the study but it is not recorded that this related in any part to behavioural reasons.
	Not neutered	There is no record of the dog being neutered during the study, either through no surgery being recorded or writing that the dog is entire or not mentioning the dog's neuter status.
Did the owner seek referred solution?	Dog trainer	The owner has been in contact with, phone or met with, a dog trainer to seek help. This includes training classes but does not include puppy socialisation classes taken before an undesirable behaviour was exhibited.

	Animal behaviourist	The owner has been in contact with, phone or met with, an Animal behaviourist to seek help.
	Veterinary advice	The owner had specifically gone to the veterinarians for a behavioural consult or called the veterinarians for the primary reason of discussing behaviour.
	Not specified	When the record states that ‘the owner tried everything’ or suggests that the owner sought help elsewhere but it does not give specifics.
	Owner did not try anything	It is not written in the EPR that the owner has tried any solution.
Did the vet advise referral?	Referral advice recorded	The veterinarian advised the owner to seek external help in the form of an animal behaviourist, dog trainer or charity. The veterinarian could additionally provide a telephone number for the owner or offer to write a referral letter.
	No referral advice recorded	The veterinarian does not provide any advice to the owner to seek external help.
Source of dog		The source of dog is recorded from the information in the EPR.
Did the owner attempt to rehome		The record was examined to see if any attempt was made to rehome the dog. It

the dog before death?		could have been to another home or to a rescue centre.

Appendix Table 3: Estimated prevalence for deaths (n = 1,574) ascribed to undesirable behaviour (UB) among dogs attending primary-care veterinary practices in England that died before three years of age with an ascribed cause.

Breed	Total	Not UB	UB	Not recorded	Prevalence of UB*
Other purebreds	326	202	79	45	28.1
Boxer	21	20	0	1	0.0
Bulldog	29	22	4	3	15.4
French Bulldog	23	19	2	2	9.5
Chihuahua	55	44	11	0	20.0
Border Collie	34	22	9	3	29.0
Crossbreed	364	192	132	40	40.7
Dogue de Bordeaux	21	17	3	1	15.0
Husky	18	11	6	1	35.3
Pug	22	19	1	2	5.0
Labrador Retriever	54	39	12	3	23.5
Rottweiler	38	21	11	6	34.4
German Shepherd Dog	57	36	14	7	28.0
Shih-tzu	40	27	10	3	27.0
Cavalier King Charles Spaniel	27	17	8	2	32.0
Cocker Spaniel	41	19	20	2	51.3
English Springer Spaniel	16	11	5	0	31.3
American Pit Bull Terrier	34	1	26	7	96.3
Jack Russell Terrier	91	49	34	8	41.0
Staffordshire Bull Terrier	191	71	96	24	57.5
West Highland White Terrier	16	7	6	3	46.2
Yorkshire Terrier	56	39	13	4	25.0
Total	1,574	905	502	167	35.7

*Prevalence calculated among dogs with an ascribed cause of death
