# Supplementary material for: Mortality Resulting from Undesirable Behaviours in Dogs Aged Three Years and under Attending Primary-Care Veterinary Practices in Australia
Source: Animals (Basel). 2021 Feb 13;11(2):493. doi: 10.3390/ani11020493 (PMC7918417; doi:10.3390/ani11020493)
Supplement: Supplementary file 1 [file animals-11-00493-s001.zip › animals-1055081-supplementary/File S2.pdf]

Breed Selection for Student Research Projects

Rationale

In order to conduct robust breed-based studies, it is important to be certain that the breeds studied are those for which we have a clear standard. For this reason, breed-specific studies will only be conducted on breeds recognised by the Australian National Kennel Club (ANKC), the Australian Cat Federation (ACF), the Department of Animal Science at Oklahoma State University (OSU) or the Royal Agricultural Society (RAS).

Breeds grouped together

In some cases there are several breeds represented within a group and one of label given is a generic breed name that may or may not be recognised by the relevant body. For any such group where a less specific breed name has been allocated to at least 66% of patients within that group, all animals have been combined into a single breed. The groups identified, along with the percentage of individuals within each that have been allocated the generic breed name, are listed here.

**Group**	**%**	**Includes:**
Chihuahua	95%	Chihuahua Long Hair; Chihuahua Short Hair; Chihuahua
Corgi	75%	Corgi; Welsh Corgi; Cardigan Welsh Corgi; Pembroke Welsh Corgi
Dachshund	99%	Long-Haired Standard Dachshund; Smooth-Haired Dachshund; Wire-Haired Dachshund; Dachshund; Long-Haired Miniature Dachshund; Smooth-Haired Miniature Dachshund; Wire-Haired Miniature Dachshund
Fox Terrier	97%	Fox Terrier; Smooth Fox Terrier; Wire-Haired Fox Terrier
Poodle (all except Standard)	76%	Poodle; Miniature Poodle; Toy Poodle
Schnauzer	66%	Schnauzer; Giant Schnauzer; Miniature Schnauzer
Donkeys	100%	Abyssinian, Anatolia, Large Standard, Mammoth, Mary, Mini, Poitou, Standard
Welsh Cob and Pony	78%	Welsh Cob; Welsh Pony; Welsh; Welsh Mountain; Welsh Section B; Welsh Section D

Collies also have multiple variants present in the database. Those labelled as “Collie” or any version not recognised by ANKC were not included or grouped together as they only represented a small percentage of all the Collies combined.

The term Rex is given in only 8% of cases of animals identified as one of the Rex breeds, whereas 75% of these are Devon Rex and 13% are Cornish Rex. As such, the breeds have been kept separate instead of being grouped together. Any simply identified as ‘Rex’ will be excluded.

Complete lists of breeds recognised by official organisations and the accepted variations of these found within the VCA database

Dogs – ANKC

ANKC Names	Accepted variations within VCA database
Affenpinscher	Affenpinscher
Afghan Hound	Afghan Hound
Airedale Terrier	Airedale Terrier
Akita	Akita
Akita (Japanese)	Akita Inu; Japanese Akita; Japanese Akita Inu
Alaskan Malamute	Alaskan Malamute; Malamute
American Staffordshire Terrier	American Staffordshire Terrier; American Staffordshire
Anatolian Shepherd Dog	Anatolian Shepherd
Australian Cattle Dog	Australian Cattle Dog; Blue Cattledog; Blue Heeler; Cattle Dog; Red Heeler
Australian Kelpie	Australian Kelpie; Kelpie
Australian Shepherd	Australian Shepherd
Australian Silky Terrier	Australia Shepherd; Australian Silky Terrier; Silky Terrier; Sydney Silky Terrier; Sydney Terrier
Australian Stumpy Tail Cattle Dog	Stumpy Tail Cattle Dog
Australian Terrier	Australian Terrier
Azawakh	Azawakh
Basenji	Basenji
Basset Fauve De Bretagne	Basset Fauve De Bretagne
Basset Hound	Basset Hound
Beagle	Beagle
Bearded Collie	Bearded Collie
Bedlington Terrier	Bedlington Terrier
Belgian Shepherd (Groen)	Belgian Shepherd Groenendael; Groenendael
Belgian Shepherd (Laek)	Belgian Laeken; Belgian Shepher Laekenois
Belgian Shepherd (Mal)	Belgian Malinois; Belgian Shepherd Malinois; Malinois
Belgian Shepherd (Terv)	Belgian Shepherd Tervuren; Belgian Tervuren
Bergamasco Shepherd Dog	Bergamasco
Bernese Mountain Dog	Bernese Mountain Dog
Bichon Frise	Bichon Frise
Bloodhound	Bloodhound
Bluetick Coonhound	Bluetick Coonhound
Border Collie	Border Collie
Border Terrier	Border Terrier
Borzoi	Borzoi
Boston Terrier	Boston Terrier
Bouvier Des Flandres	Bouvier Des Flandres
Boxer	Boxer
Bracco Italiano	Bracco Italiano
Briard	Briard
British Bulldog	British Bulldog
Brittany	Brittany; Brittany Spaniel; French Brittany Spaniel
Bull Terrier	Bull Terrier
Bull Terrier (Miniature)	Miniature Bull Terrier

Bullmastiff	Bull Mastiff
Cairn Terrier	Cairn Terrier
Canaan Dog	Canaan Dog
Canadian Eskimo Dog	Canadian Eskimo Dog
Cane Corso	Cane Corso
Cavalier King Charles Spaniel	Cavalier King Charles Spaniel
Central Asian Shepherd Dog	Central Asian Shepherd
Cesky Terrier	Cesky Terrier
Chesapeake Bay Retriever	Chesapeake Bay Retriever
Chihuahua GROUPED	Chihuahua Long Hair; Chihuahua Short Hair; Chihuahua
Chinese Crested Dog	Chinese Crested (Hairless); Chinese Crested Powderpuff
Chow Chow	Chow Chow
Clumber Spaniel	Clumber Spaniel
Cocker Spaniel	Cocker Spaniel
Cocker Spaniel (American)	American Cocker Spaniel
Collie (Rough)	Collie (Rough); Rough Collie
Collie (Smooth)	Collie (Smooth); Smooth Collie
Corgi GROUPED	Corgi; Welsh Corgi; Cardigan Welsh Corgi; Pembroke Welsh Corgi
Coton De Tulear	Coton De Tulear
Curly-Coated Retriever	Curly-Coated Retriever
Dachshund GROUPED	Long-Haired Standard Dachshund; Smooth-Haired Dachshund; Wire-Haired Dachshund; Dachshund; Long-Haired Miniature Dachshund; Smooth-Haired Miniature Dachshund; Wire-Haired Miniature Dachshund
Dalmatian	Dalmatian
Dandie Dinmont Terrier	Dandie Dinmont Terrier
Deerhound	Deer Hound
Dobermann	Doberman; Doberman Pinscher
Dogue De Bordeaux	Bordeaux Bulldog; Dogue De Bordeaux; French Mastiff
Dutch Shepherd	Dutch Shepherd Dog
English Setter	English Setter
English Springer Spaniel	English Springer Spaniel
English Toy Terrier (Black & Tan)	English Toy Terrier
Estrela Mountain Dog	Estrela Mountain Dog
Eurasier	Eurasier
Field Spaniel	Field Spaniel
Finnish Lapphund	Finnish Lapphund
Finnish Spitz	Finnish Spitz
Flat Coated Retriever	Flat-Coated Retriever
Fox Terrier GROUPED	Fox Terrier; Smooth Fox Terrier; Wire-Haired Fox Terrier
Foxhound	Foxhound
French Bulldog	French Bulldog
German Hunting Terrier	German Hunt Terrier
German Pinscher	German Pinscher
German Shepherd Dog	German Shepherd
German Shorthaired Pointer	German Short-Haired Pointer
German Spitz	German Spitz
German Wirehaired Pointer	German Wire-Haired Pointer
Glen Of Imaal Terrier	Glen Of Imaal Terrier

Golden Retriever	Golden Retriever
Gordon Setter	Gordon Setter
Grand Basset Griffon Vendeen	Grand Griffon Vendeen
Great Dane	Great Dane
Greyhound	Greyhound
Griffon Bruxellois	Brussels Griffon; Bruxellois Griffon; Belgian Griffon
Hamiltonstovare	Hamiltonstovare; Hamiltonstøvare
Harrier	Harrier
Havanese	Havanese
Hungarian Vizsla	Hungarian Vizsla
Hungarian Wirehaired Vizsla	Wire Haired Vizsla
Ibizan Hound	Ibizan Hound
Icelandic Sheepdog	Icelandic Sheepdog
Irish Red & White Setter	Irish Red & White Setter
Irish Setter	Irish Setter
Irish Terrier	Irish Terrier
Irish Water Spaniel	Irish Water Spaniel
Irish Wolfhound	Irish Wolfhound
Italian Greyhound	Italian Greyhound
Italian Spinone	Italian Spinone
Italian Spinone	Spinone Italiano
Jack Russell Terrier	Jack Russell Terrier
Japanese Chin	Japanese Chin; Japanese Spaniel
Japanese Spitz	Japanese Spitz
Keeshond	Keeshond
Kerry Blue Terrier	Kerry Blue Terrier
King Charles Spaniel	King Charles Spaniel
Komondor	Komondor
Kuvasz	Kuvasz; Hungarian Kuvasz
Labrador Retriever	Labrador Retriever; Labrador
Lagotto Romagnolo	Lagotto Romagnolo; Lagotto
Lakeland Terrier	Lakeland Terrier
Large Munsterlander	Large Munsterlander; Large Münsterländer
Leonberger	Leonberger
Lhasa Apso	Lhasa Apso
Lowchen	Lowchen
Maltese	Maltese; Maltese Terrier
Manchester Terrier	Manchester Terrier
Maremma Sheepdog	Maremma Sheepdog
Mastiff	Mastiff
Miniature Pinscher	Miniature Pinscher; Miniature Doberman Pinscher
Neapolitan Mastiff	Neapolitan Mastiff
Newfoundland	Newfoundland
Norfolk Terrier	Norfolk Terrier
Norwegian Buhund	Norwegian Buhund
Norwegian Elkhound	Norwegian Elkhound
Norwich Terrier	Norwich Terrier
Nova Scotia Duck Tolling Retriever	Nova Scotia Duck-Tolling Retriever

Old English Sheepdog	Old English Sheepdog
Otterhound	Otterhound
Papillon	Papillon
Parson Russell Terrier	Parson Russell Terrier
Pekingese	Pekingese
Peruvian Hairless Dog	Peruvian Hairless Dog
Petit Basset Griffon Vendeen	Petit Basset Griffon Vendeen
Pharaoh Hound	Pharaoh Hound
Pointer	Pointer
Polish Lowland Sheepdog	Polish Lowland Sheepdog
Pomeranian	Pomeranian
Poodle (except Standard) GROUPED	Poodle; Miniature Poodle; Toy Poodle
Poodle (Standard)	Standard Poodle
Portuguese Podengo	Portuguese Podengo
Portuguese Water Dog	Portuguese Water Dog
Pug	Pug
Puli	Puli; Hungarian Puli
Pyrenean Mastiff	Pyrenean Mastiff
Pyrenean Mountain Dog	Pyrenean Mountain Dog
Pyrenean Sheepdog Longhaired	N/A
Rhodesian Ridgeback	Rhodesian Ridgeback
Rottweiler	Rottweiler
Russian Black Terrier	Black Russian Terrier
Russian Toy	Russian Toy Terrier
Saluki	Saluki
Samoyed	Samoyed
Schipperke	Schipperke
Schnauzer GROUPED	Schnauzer; Giant Schnauzer; Miniature Schnauzer
Scottish Terrier	Scottish Terrier
Sealyham Terrier	Sealyham Terrier
Shar pei	Chinese Shar-Pei; Shar-Pei
Shetland Sheepdog	Shetland Sheepdog
Shiba Inu	Japanese Shiba Inu; Shiba Inu
Shih Tzu	Shih Tzu
Siberian Husky	Siberian Husky; Husky
Skye Terrier	Skye Terrier
Sloughi	Sloughi
Soft Coated Wheaten Terrier	Irish Soft Coated Wheaten Terrier; Irish Soft-Coated Wheaten Terrier; Soft-Coated Wheaten Terrier
Spanish Mastiff	Spanish Mastiff
Spanish Water Dog	Spanish Water Dog
St. Bernard	Saint Bernard; St. Bernard
Staffordshire Bull Terrier	Staffordshire Bull Terrier
Sussex Spaniel	Sussex Spaniel
Swedish Lapphund	Swedish Lapphund
Swedish Vallhund	Swedish Vallhund
Tenterfield Terrier	Tenterfield Terrier
Tibetan Mastiff	Tibetan Mastiff
Tibetan Spaniel	Tibetan Spaniel

Tibetan Terrier	Tibetan Terrier
Weimaraner	Weimaraner
Welsh Springer Spaniel	Welsh Springer Spaniel
Welsh Terrier	Welsh Terrier
West Highland White Terrier	West Highland White Terrier
Whippet	Whippet
Xoloitzcuintle	Xoloitzcuintle
Yorkshire Terrier	Yorkshire Terrier

Cats – ACF

ACF Names	Accepted variations within VCA database
Abyssinian	Abyssinian
American Curl (Shorthair and Longhair)	American Curl
American Shorthair	American Short Hair
Aphrodite (Longhair and Shorthair)	N/A
Australian Mist	Australian Mist; Spotted Mist
Balinese	Balinese
Bengal	Bengal
Birman	Birman
Bombay	Bombay
British Shorthair	British Short Hair; British Blue
Burmese	Burmese; Burmese, European(?)
Burmese (American)	N/A
Burmilla (Shorthair and Longhair)	Burmilla; Longhair Burmilla
Cornish Rex	Cornish Rex
Cymric (Longhaired Manx)	Cymric; Long Hair Manx
Devon Rex	Devon Rex
Egyptian Mau	Egyptian Mau
Exotic	Exotic Long Hair; Exotic Short Hair
Foreign White (Shorthair and Longhair)	Foreign White
Japanese Bobtail (Shorthair and Longhair)	Japanese Bobtail
Korat	Korat
Laperm (Shorthair and Longhair)	Laperm, Long Hair; Laperm, Short Hair
Lykoi	N/A
Maine Coon	Maine Coon
Mandalay	N/A
Manx	Manx
Neva Masquerade	N/A
Norwegian Forest Cat	Norwegian Forest
Ocicat	Ocicat
Oriental Longhair (formerly Javanese)	Oriental Long Hair; Javanese
Oriental Shorthair	Oriental Short Hair; Oriental
Persian	Persian; Chinchilla
Peterbald	Peterbald
Pixiebob (Shorthair and Longhair)	Pixiebob
Ragdoll	Ragdoll
Russian	Russian Black; Russian Blue; Russian White
Scottish Fold (Shorthair and Longhair)	Scottish Fold

Scottish Shorthair/Scottish Longhair	Scottish Shorthair; Scottish Straight
Selkirk Rex (Shorthair and Longhair)	Selkirk Rex
Siamese	Siamese
Siberian	Siberian
Singapura	Singapura
Snowshoe	Snowshoe
Somali	Somali
Sphynx	Sphynx
Tonkinese	Tonkinese
Turkish Angora	Turkish Angora
Turkish Van	Turkish Van

Horses – OSU or RAS

OSU or RAS	Currently in VCA database
(several breeds)	Donkey
Andalusian	Andalusian
Appaloosa	Appaloosa
Arabian	Arab
Australian Brumby	Australian Brumby
Australian Stock Horse	Australian Stockhorse
Australian Stockhorse (RAS)	Stockhorse
Cleveland Bay	Cleveland Bay
Clydesdale	Clydesdale
Connemara Pony	Connemara
Dartmoor Pony	Dartmoor
Friesian	Friesian
Hackney	Hackney
Haflinger	Haflinger
Hanoverian	Hanoverian
Irish Draught	Irish Draught
Lipizzan	Lippazan
Miniature	Miniature Horse
Morgan	Morgan
New Forest Pony	New Forest
Paint	Paint
Palomino	Palomino
Percheron	Percheron
Quarter Horse	Quarterhorse
Saddlebred	Saddlebred
Shetland Pony	Shetland
Shire	Shire
Standardbred	Standardbred
Thoroughbred	Thoroughbred
Trakehner	Trakehner
Welsh Pony and Cob	Welsh Cob; Welsh Pony; Welsh; Welsh Mountain; Welsh Section B; Welsh Section D

Version control

Version No.	Date	Name	Details
Version 1.0	5/6/19	Sophie Masters	Original document outlining breed selection rationale and guidelines.
